# The role of pancreatoscopy in the diagnostic work-up of intraductal papillary mucinous neoplasms: a systematic review and meta-analysis

**DOI:** 10.1055/a-1869-0180

**Published:** 2022-07-20

**Authors:** David M. de Jong, Pauline M. C. Stassen, Bas Groot Koerkamp, Mark Ellrichmann, Petko I. Karagyozov, Andrea Anderloni, Leena Kylänpää, George J. M. Webster, Lydi M. J. W. van Driel, Marco J. Bruno, Pieter J. F. de Jonge, on behalf of the European Cholangioscopy study group

**Affiliations:** 1 Department of Gastroenterology and Hepatology, Erasmus MC University Medical Center Rotterdam, Rotterdam, The Netherlands; 2 Department of Surgery, Erasmus MC Cancer Institute, Erasmus MC University Medical Center Rotterdam, Rotterdam, The Netherlands; 3 Department of Interdisciplinary Endoscopy, University Medical Center Schleswig-Holstein, Campus Kiel, Kiel, Germany; 4 Department of Interventional Gastroenterology, Acibadem City Clinic Tokuda University Hospital, Sofia, Bulgaria; 5 Digestive Endoscopy Unit, Department of Gastroenterology, Humanitas Clinical and Research Center, IRCCS, Milan, Italy; 6 Abdominal Center, Gastroenterological Surgery, Helsinki University Hospital and University of Helsinki, Helsinki, Finland; 7 Department of Gastroenterology, University College London Hospitals, London, UK; 8 Sheila Sherlock Liver Centre, Royal Free Hospital, London, UK

## Abstract

**Background** Confirming the diagnosis, invasiveness, and disease extent of intraductal papillary mucinous neoplasms (IPMNs) of the pancreas is challenging. The aim of this study was to summarize the literature on the efficacy and safety of peroral pancreatoscopy (POP) in the diagnosis of IPMN, including the impact of pre- and intraoperative POP on the management of IPMN.

**Methods** The EMBASE, Medline Ovid, Web of Science, Cochrane CENTRAL, and Google Scholar databases were systematically searched for articles. Eligible articles investigated cohorts of patients who underwent POP for (suspected) IPMN.

**Results** 25 articles were identified and included in this review; with 22 of these reporting on the diagnostic yield of POP in IPMN and 11 reporting on the effect of pre- or intraoperative POP on clinical decision-making. Cannulation and observation rates, and overall diagnostic accuracy were high across all studies. Frequently reported visual characteristics of IPMN were intraductal fish-egg-like lesions, hypervascularity, and granular mucosa. Overall, the adverse event rate was 12 %, primarily consisting of post-endoscopic retrograde cholangiopancreatography pancreatitis, with a pooled rate of 10 %, mostly of mild severity. Regarding the impact of POP on clinical decision-making, POP findings altered the surgical approach in 13 %–62 % of patients.

**Conclusion** POP is technically successful in the vast majority of patients with (suspected) IPMN, has a consistently high diagnostic accuracy, but an adverse event rate of 12 %. Data on intraoperative pancreatoscopy are scarce, but small studies suggest its use can alter surgical management. Future studies are needed to better define the role of POP in the diagnostic work-up of IPMN.

## Graphical abstract

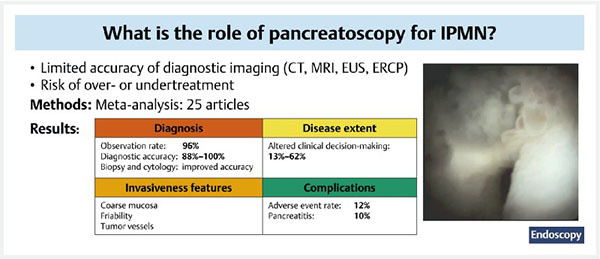

## Introduction

Intraductal papillary mucinous neoplasm (IPMN) is a common precancerous lesion of the pancreas, characterized by intraductal papillary proliferation of mucin-producing cells, resulting in cystic dilatation of the pancreatic duct (PD) 1 2 . IPMN may progress from adenomatous lesions to high grade dysplasia (HGD) and finally to invasive carcinoma. Branch-duct IPMN (BD-IPMN) is the most prevalent subtype. Main-duct IPMN (MD-IPMN) is however associated with the highest risk of progression to malignancy and is considered an indication for surgery if the main PD (MPD) diameter is > 10 mm, or if there is evidence of jaundice or mural nodules 3 4 . Early identification of MD-IPMN is important to allow surgery to be performed before the development of cancer.

Currently, imaging modalities used for the diagnosis of IPMN include computed tomography (CT), magnetic resonance imaging (MRI), and endoscopic ultrasonography (EUS). Even with the addition of endoscopic retrograde cholangiopancreatography (ERCP) and ERCP-guided brushing, the diagnosis of MD-IPMN and BD-IPMN can be challenging, as is determining the presence of HGD or invasive carcinoma. As a result, some patients undergo unnecessary pancreatic surgery for IPMN with low grade dysplasia (LGD) or benign cystic lesions 5 . In addition, when surgery is indicated, preoperative determination of the extent of MD-IPMN using these techniques can be difficult. This could result in either unnecessary loss of pancreatic tissue in the case of an overly extensive resection, or progression of disease in the case of an incomplete resection. Although intraoperative frozen section analysis of the resection margin is routinely performed during pancreatic surgery, this strategy does not account for discontinuous “skip” lesions 6 .

Over the past decades, peroral pancreatoscopy (POP) has been used more frequently in the diagnostic work-up of pancreaticobiliary disorders. It has potential additional value for the diagnosis of IPMN and in determining the intraductal extent of the lesion. Moreover, intraoperative pancreatoscopy (IOP) can assess residual skip lesions 7 . However, the exact role of POP in the diagnosis and treatment of IPMN is unclear. Therefore, this systematic review and meta-analysis aimed to summarize the current literature on the technical success, safety, diagnostic yield, and clinical utility of POP in the management of IPMN.

## Methods

### Eligibility criteria

Studies eligible for inclusion were randomized controlled trials, prospective and retrospective cohort studies, and case series. Case reports, reviews, poster abstracts, and studies in a language other than English were excluded. Studies examining adults with a (suspected) diagnosis of IPMN, undergoing POP, either performed during diagnostic work-up for IPMN or perioperatively, were deemed eligible.

### Search strategy and study selection

On 11 February 2022, according to the PRISMA guidelines ( **Table 1 s** , see online-only Supplementary material), a systematic literature search was performed in EMBASE, Medline Ovid, Web of Science, Cochrane CENTRAL, and Google Scholar. Predefined keywords used in this search were “pancreatoscopy” and “IPMN” to identify relevant articles. The full search strategy is presented in **Table 2 s** .

After duplicates of the retrieved articles had been removed, the titles and abstracts were independently screened for eligibility by two authors (D.d.J. and P.S.). The full text of potentially relevant articles was retrieved and independently assessed. Disagreement was resolved by consensus after discussion with a third author (P.J.d.J.). The references listed within the selected articles were screened to identify additional studies relevant for inclusion in this literature review.

### Data extraction

Data were systematically extracted from all included studies using a predefined standardized form. Data extracted included study design, patient characteristics, and intervention-related characteristics (e. g. successful cannulation and the ability to visualize the target area). In addition, any pancreatoscopic visual characteristics of IPMN that were reported in the articles, the use and diagnostic value of adjunctive modalities such as NBI, and the effect of POP findings on clinical management were noted.

The evaluated outcomes were: (i) technical success, defined as the ability to advance the pancreatoscope to the target area/lesion within the MPD, and safety, including adverse events (AEs) such as post-ERCP pancreatitis (PEP), perforation, and bleeding; (ii) diagnostic pancreatoscopic features and accuracy, defined as the rate of agreement between these features and pathological examination of the surgical or autopsy specimens, for both POP visualization alone and for POP-guided biopsy or cytology; and (iii) the effect on clinical decision-making, defined as the surgical approach being altered on the basis of the pre- or intraoperative pancreatoscopic findings.

### Statistical analysis

Statistical analyses were mostly limited to descriptive statistics using frequencies and percentages. For the pooled AE rate, meta-analysis was performed using R version 4.0.1 with the “meta” package. Random-effects meta-analysis was used, regardless of the results of heterogeneity testing. The effect size can vary per study because of differences in cohorts and the included patients, therefore random-effect meta-analysis is more suitable than fixed-effects meta-analysis. Data are presented as means with 95 % CIs. Sensitivity analysis was performed for studies that reported the AEs on patients with IPMN only and for all patients, also including patients who underwent POP for indications other than IPMN.

## Results

**Fig. 1:**
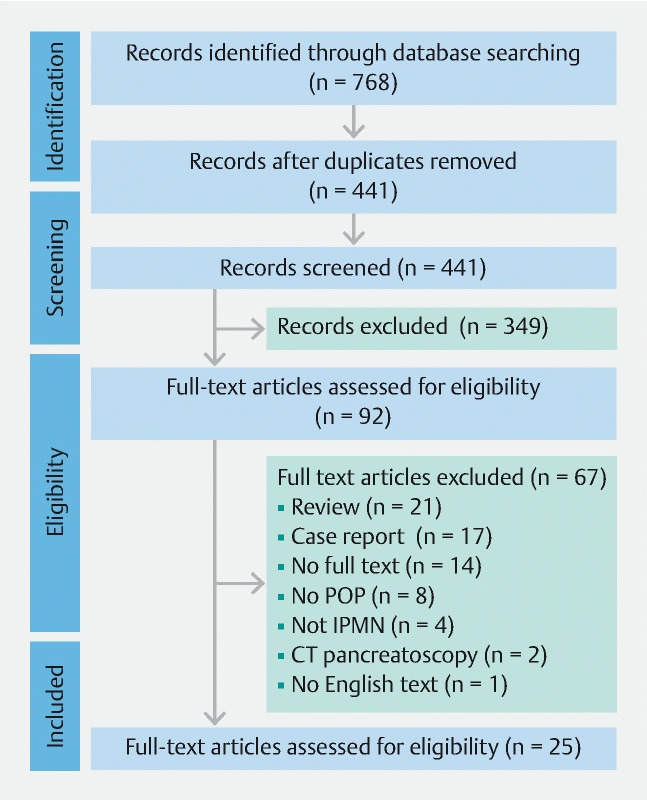
Flowchart showing the selection and exclusion of articles. POP, peroral pancreatoscopy; IPMN, intraductal papillary mucinous neoplasm; CT, computed tomography.

A total of 25 articles met the inclusion criteria 8 9 10 11 12 13 14 15 16 17 18 19 20 21 22 23 24 25 26 27 28 29 30 31 32 . The process of article selection and reasons for exclusion are summarized in Fig. 1 . The data extracted from the included studies are summarized in Table 1 and Table 2 . There were 22 studies that primarily reported on the diagnostic yield of POP for all IPMN types 8 9 10 11 12 13 14 15 16 17 18 19 21 22 24 25 26 27 28 29 30 32 and 11 articles that reported on the effect of the POP findings, in the preoperative and/or intraoperative setting, on clinical decision-making (i. e. choice to proceed to surgery and extent of surgery) 10 13 14 19 21 22 23 24 28 29 31 .

**Table 1:** Studies investigating the role of peroral pancreatoscopy in the diagnosis and treatment of intraductal papillary mucinous neoplasm (IPMN).

Author (year)	Design (n)	Cannulations/target area observed, %	Pancreatoscopy type	Key findings	Adjunct modalities	AE, %
Fujita et al. (1990) 8	Retro (8)	86/100	Mother–baby: PF24, XCPF 3.3, BF-3C10, CHF-P10	IOP is useful for determination of lesion extent	NA	NA
Özkan et al. (1995) 9	Retro (9)	89/100	Mother–baby: CFS-B20SL	IPMN is characterized by villous-like mucosal growths and clear jelly-like mucin substances	NA	0
Kaneko et al. (1998) 10	Prosp (24)	100/100	Ultrathin pancreatoscope	IOP is able to detect lesions not detected by preoperative ERCP or EUS	NA	NA
Mukai et al. (1998) 11	Retro (25)	100/60	Mother–baby: CHF-B20, CHF-B200, CHF-BP30, XCHF-B27	Papillary lesions > 3 mm have a higher chance of malignancy	Biopsy: sensitivity 57 %, specificity 100 %, accuracy 75 % Irrigation fluid cytology: sensitivity 63 %, specificity 100 %, accuracy 89 %	4
Yamaguchi et al. (2000) 12	Retro (41)	100/73	Mother–baby: XCHF-B27, CHF-B20, CHF-BP30	Severe atypical adenoma and carcinoma are associated with multiple morphologic features (i. e. frequently villous or vegetative elevations and red colored markings)	NA	NA
Atia et al. (2002) 13	Retro (5)	100/100	FCP-9P	100 % correct diagnosis of IPMN by POP	NA	20
Hara et al. (2002) 14	Retro (60)	100/100	Mother–baby: CHF-BP30, OPTISCOPE	Combination of IDUS and POP improve differentiation between malignant and benign IPMN	Pancreatic juice cytology: sensitivity 13 %, specificity 100 %, accuracy 44 % IDUS (MD): sensitivity 56 %, specificity: 71 %, accuracy 63 % IDUS (BD): sensitivity: 77 %, specificity: 100 %, accuracy 88 %	6.7
Yamao et al. (2003) 15	Retro (60)	95/100	Mother–baby: CPF-PAB, PF8	Friability and protruding lesions more frequently seen in malignancy	NA	12 1
Yamaguchi et al. (2005) 32	Retro (103)	100/100	Mother–baby: CHF-BP30	Cytology has better diagnostic accuracy when collected by POP than when catheter-assisted	Pancreatic juice cytology: sensitivity 68 %, specificity 100 %	NA
Yasuda et al. (2005) 16	Retro (26)	100/100	Mother–baby: NA	Detection of polypoid tumor > 3 mm by POP 67 % No adenocarcinoma in protrusions < 3 mm	Biopsy: sensitivity 50 %, specificity 100 % Pancreatic juice cytology: sensitivity 50 %, specificity 100 %	0
Itoh et al. (2007) 17	Prosp (5)	100/100	Mother–baby: CHF-BP260	NBI improves visualization of small vessels and superficial architecture	NBI	NA
Itoi et al. (2007) 18	Retro (3)	100/100	Mother–baby: CHF-BP260	NBI is able to identify skip lesions otherwise not seen and shows capillary vessels more clearly	NBI	0
Miura et al. (2010) 19	Prosp (21)	100/91	Mother–baby: CHF-BP260, CHF-B260	POP combined with NBI shows vascular patterns and protrusions more clearly and is useful for differentiation	NBI: correct excision line based on POP + NBI in 100 % patients	0
Brauer et al. (2013) 20	Retro (4)	100/100	Mother–baby: CHF-BP30 or Spyglass DVS	POP via dorsal duct is technically feasible	NA	0
Arnelo et al. (2014) 21	Prosp (41)	93/100	SpyGlass DVS	Overall: sensitivity 84 %, specificity 75 % Accuracy: for MD-IPMN 76 %, for BD-IPMN 78 %	Biopsy in 17/41: benign 9, HGD 4, inadequate 4 Irrigation fluid cytology in 22/41: malignancy 5 %	17
Nagayoshi et al. (2014) 22	Retro (17)	77/100	SpyGlass DVS or ERCP catheter pancreatoscopy	100 % sensitivity of irrigation fluid cytology for detecting malignancy	Biopsy: sensitivity 25 %, specificity 100 % Irrigation fluid cytology: sensitivity 100 %, specificity 100 %	35
Pucci et al. (2014) 23	Retro (18)	100/100	Flexible choledochoscope	IOP is a valuable tool to determine the surgical resection margin	NA	NA
Navez et al. (2014) 24	Retro (21)	100/100	NA	IOP is able to detect occult lesions and in combination with biopsies it could change the initial surgical plan	NA	NA
Kurihara et al. (2016) 25	Prosp (17)	88/100	SpyGlass DVS	Visual diagnostic accuracy of POP in MD-IPMN was 87.5 % (i. e. papillary stricture, fish-egg-like lesion)	Biopsy: 91 % adequate samples	0
El Hajj et al. (2017) 26	Retro (78)	100/97	Mother–baby: CHF-BP30, CHF-BP160, CHF-Y002 or SpyGlass DVS	POP-directed biopsies increased diagnostic accuracy of visual impression	Biopsy: sensitivity 87 %, specificity 100 %, PPV 100 %, NPV 84 %, accuracy 92 % (in differentiating between neoplasia and non-neoplasia)	12 2
Parbhu et al. (2017) 27	Retro (16)	100/100	SpyGlass DVS and DS	Accuracy of biopsy alone (64 %) increased to 100 % in combination with visualization to correctly diagnose IPMN	Biopsy: sensitivity 64 %, specificity 100 % (in diagnosing IPMN)	6
Ohtsuka et al. (2018) 28	Retro (7)	100/100	SpyGlass DS	Good visualization of the target area in all patients, with low diagnostic accuracy of targeted biopsies for detecting HGD	Biopsy: sensitivity 0 % Irrigation fluid cytology: sensitivity 33 %	14
Trindade et al. (2018) 29	Retro (31)	100/100	SpyGlass DS	POP is of added value in patients with MD-IPMN and a diffusely dilated MPD, without focal lesions on cross-sectional imaging or EUS	Biopsy in 28 /31: LGD 79 %, HGD 18 %, adenocarcinoma 4 %	29
Han et al. (2019) 30	Retro (13)	100/100	Mother–baby: CHF-BP30 or SpyGlass DS	In patients with presumed idiopathic chronic pancreatitis, POP is able to identify MD-IPMN	NA	NA
Tyberg et al. (2019) 31	Retro (13)	100/100	SpyGlass DS	POP can be effectively used as mapping tool preoperatively	NA	0

AE, adverse events; BD, branch duct; ERCP, endoscopic retrograde cholangiopancreatography; EUS, endoscopic ultrasonography; HGD, high grade dysplasia; IDUS, intraductal ultrasonography; IOP, intraoperative pancreatoscopy; MD, main duct; MPD, main pancreatic duct; NBI, narrow-band imaging; NPV, negative predictive value; NA, not applicable; POP, peroral pancreatoscopy; PPV, positive predictive value; Prosp, prospective; Retro, retrospective.

1 Calculated over the total group of patients and not in patients with IPMN only.

2 Calculated per pancreatoscopy procedure, not per patient.

**Table 2:** Studies investigating the effect of preoperative or intraoperative pancreatoscopy on clinical management of intraductal papillary mucinous neoplasm (IPMN).

Author (year)	Design (n)	Timing	Gold standard	Adjunctive modality	Excision line based on pancreatoscopic findings	Altered surgical approach
Kaneko et al. (1998) 10	Prosp (24)	IOP	Surgical specimen	NA	NA	13 %: 10/24 extra lesions detected; 5/10 multifocal lesions; 3 /5 more extensive resection
Atia et al. (2002) 13	Retro (5)	Preop	Surgical specimen	NA	In 4/4 of patients with IPMN (100 %); pancreatic cyst identified in fifth patient	NA
Hara et al. (2002) 14	Retro (40)	Preop	Surgical specimen	IDUS	Continuous lesion in 35/40 (87.5 %). Positive resection margin in one patient (2.5 %)	NA
Miura et al. (2010) 19	Retro (21)	Preop	Surgical specimen	NBI	In 7/7 patients, negative resection margins	
Arnelo et al. (2014) 21	Prosp (44)	Preop	Surgical specimen, follow-up	NA	NA	95 % additional information, in 76 % affected clinical decision-making
Nagayoshi et al. (2014) 22	Retro (17)	Preop	Radiology, surgical specimen	NA	In three patients (17.6 %), excision line determined	
Pucci et al. (2014) 23	Retro (18)	IOP	Surgical specimen	NA	NA	33 %: 29 % extended margins; 6 % spared margins
Navez et al. (2015) 24	Retro (21)	IOP	Radiology, surgical specimen	NA	NA	Additional lesions detected in 38 %: 29 % extended margins, 6 % spared margins
Ohtsuka et al. (2018) 28	Retro (7)	Preop	Surgical specimen	NA	NA	14 % extended margins
Trindade et al. (2018) 29	Retro (31)	Preop	Surgical specimen	NA	Surgery dictated by POP on basis of additional findings in 42 %; significantly more often in patients with a diffusely dilated MPD (77 % vs. 17 %, *P* = 0.001)	NA
Tyberg et al. (2019) 31	Retro (13)	Preop	Surgical specimen	NA	NA	62 %: 31 % extended margins, 31 % spared margins Positive resection margins in 2/4, with spared margins (50 %)

IDUS, intraductal ultrasonography; IOP, intraoperative pancreatoscopy; NA, not applicable; NBI, narrow-band imaging; Prosp, prospective; MPD, main pancreatic duct; Preop, preoperative; Retro, retrospective.

### Technical success and adverse events

In all studies, technical success rates were reported, defined as the ability to advance the pancreatoscope into the MPD. This cannulation rate ranged from 86 % to 100 % 8 9 10 11 12 13 14 15 16 17 18 19 20 21 22 23 24 25 26 27 28 29 30 31 32 . Pancreatoscopy was performed for diagnosis of a suspected MD-IPMN, BD-IPMN, or mixed-type IPMN (MT-IPMN). A dilated MPD was not required. Predictive factors reported for successful cannulation were a dilated MPD or a wide papillary orifice. It was not often reported how many patients underwent papillotomy prior to or during the POP procedure; however, where reported, it ranged from 0 % to 92.7 %. One study reported that POP was successfully performed via the minor papilla in four patients 20 .

After successful cannulation, the rate of adequate visualization of the PD and region of interest ranged from 60 % to 100 %, but the specific location of the lesion in the PD was often not described. Of the 25 included studies, only six reported an observation rate lower than 100 % 11 12 19 22 25 26 , resulting in a combined observation rate of 95.6 %. Reasons for the inability to visualize the target area were: inadequate clearance of mucus; a nondilated MPD; and concomitant anatomical features, such as a ductal stricture. Abundant mucus impaired visual characterization of the IPMN or wall of the MPD, despite flushing 12 25 . Visualization of BD-IPMN was more difficult as compared with MD-IPMN, mainly owing to more difficult angulation of the pancreatoscope in reaching the area of interest and the smaller diameter of the MPD 11 12 22 .

**Fig. 2:**
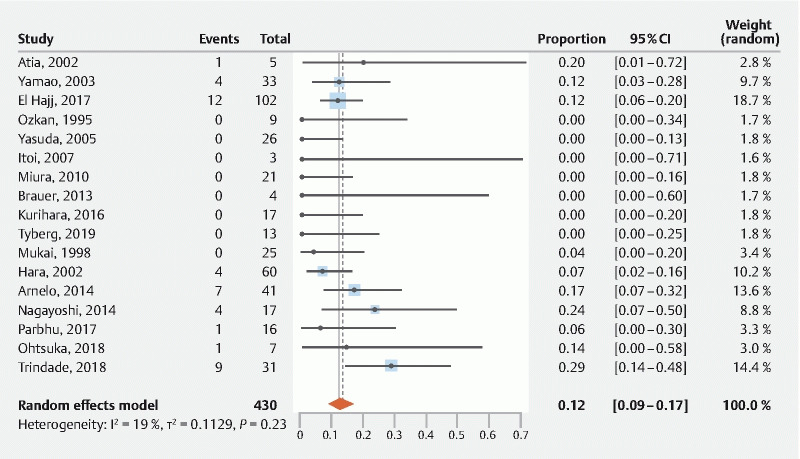
Pooled AE rate for all studies that reported adverse events.

The occurrence of AEs was reported in 17 out of 25 studies 9 11 13 14 15 16 18 19 20 21 22 25 26 27 28 29 31 . The overall pooled AE rate of these 17 studies was 12 % (95 %CI 9 %–17 %) ( Fig. 2 ). PEP was the most common AE, with a pooled incidence rate of 10 % (95 %CI 7 %–15 %) 11 13 14 15 20 21 22 26 27 28 29 ( **Fig. 1 s** ). The severity of PEP was mild in 24 patients (70.6 %), moderate in seven (20.6 %), severe in two (5.9 %), and unknown in one (2.9 %). One patient with severe PEP died 21 .

In reporting AE rates, three studies did not make a difference between patients with and without (suspected) IPMN and therefore the reported overall AE rate and PEP rate might differ for patients with IPMN, albeit sensitivity analysis showed no significant difference between these 15 20 26 . Most studies did not elaborate on the MPD diameter in relation to PEP. Trindade et al. reported that PEP occurred more frequently in patients with a focally dilated MPD (n = 7/18; 39 %) compared with patients with a diffusely dilated MPD (n = 1/13; 8 %; *P* = 0.05) 29 . In addition, Arnelo et al. reported that 6/7 patients diagnosed with PEP had a normal or only slightly dilated MPD 21 . Other AEs included post-sphincterotomy bleeding (1.3 %) 26 , a mild sedation-related event (3.2 %) 29 , and cholangitis (8.3 %) 22 . The included articles did not clarify whether surgery was deferred or postponed because of these AEs.

### Visual diagnosis of IPMN and detection of high risk features by POP



**Video 1** A clear demarcation of the proximal and distal margins of a suspected main-duct intraductal papillary mucinous neoplasm (IPMN) is shown in a case that proved to be 4-cm mixed-type IPMN without any malignancy at pancreatoduodenectomy.


**Video 2** A main-duct IPMN is delineated and peroral pancreatoscopy-guided biopsies are taken, which subsequently showed an IPMN without dysplasia, while at surgery, a main duct IPMN with low grade dysplasia was identified.


**Fig. 3:**
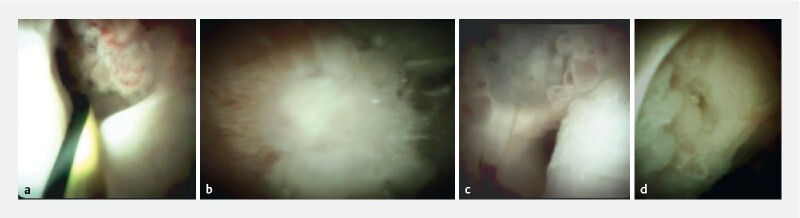
Example images during peroral pancreatoscopy (POP) in four patients with intraductal papillary mucinous neoplasm (IPMN) showing: **a** a clear proximal margin of a main-duct IPMN (MD-IPMN) that was suspicious for malignancy, but was found to be a mixed-type IPMN without any malignancy on pancreatoduodenectomy (see also Video 1 ); **b** a clear image of a visible polypoid lesion in the setting of MD-IPMN, with biopsy revealing focal malignant transformation; **c** the clear fish-egg-like lesions in an MD-IPMN; **d** a very wide side branch in the body of the pancreas, with a nodular mass seen at the opening of the side branch, which showed mild dysplasia on POP-guided biopsy and later pancreatoduodenectomy.

Pancreatoscopic characteristic features of IPMN were intraductal papillary or villous projections, and the presence of mucus 33 . Other features included: intraductal fish-egg-like lesions, that were sometimes seen on a protruding lesion, and granular mucosa 8 9 11 12 13 14 15 16 18 19 22 24 25 26 28 29 30 . However, not all classical features are consistently seen. For example, in patients with a radiological diagnosis of MD-IPMN, the classical features of IPMN, such as a fish-eye papilla and oozing of mucus from the papilla, were detected in only 35 % of patients in whom MD-IPMN was confirmed by histology 21 . Examples of some of the visual characteristics of IPMN seen on POP can be found in Fig. 3 and Video 1 and Video 2 .

Seven studies reported on the sensitivity, specificity, and overall diagnostic accuracy rates of POP in the visual diagnosis of IPMN 10 13 16 21 25 27 30 . In these studies, the results of POP were compared to pathology results after resection or from biopsies, or the absence of progression during long-term follow-up. Reported sensitivity rates ranged between 64 % and 100 % 10 13 16 21 27 30 , specificity rates between 75 % and 100 % 13 21 27 30 , and overall diagnostic accuracy between 87.5 % and 100 % 10 13 25 .

The ability to differentiate noninvasive and malignant MD-IPMN with POP has been investigated in eight studies 8 10 11 12 14 15 16 26 . The visual classification system proposed by Hara et al. allowed for discrimination of malignant IPMN from noninvasive IPMN with an accuracy of 88 % for MD-IPMN and 67 % for BD-IPM 14 . Pancreatoscopic findings that were more frequently observed in patients diagnosed with malignancy were a coarse mucosa, friability, and tumor vessels 12 15 16 . Another important finding by Trindade et al. was that 13 patients (42 %) had additional high risk features on POP that were not seen on imaging or EUS, for example papillary projections, nodules, and in one patient a frank mass 29 .

Additional imaging modalities, such as narrow-band imaging (NBI) using the Olympus CHF-BP260, have been evaluated in three studies 17 18 19 and are described in **Appendix 1 s** . The diagnostic value of targeted biopsies, cytology, and pancreatic juice collection are presented in Table 1 and **Appendix 2 s** .

### Effect on clinical decision-making

Eleven articles reported on the pre- or intraoperative assessment of the intraductal extent of IPMN by pancreatoscopy and the effect of its findings on clinical management 10 13 14 19 21 22 23 24 28 29 31 .

In four studies, the resection line was based on preoperative POP findings 13 14 19 29 . Atia et al. reported that POP correctly identified and located IPMN in 4/4 patients with a final diagnosis of IPMN (100 %) and correctly identified a pancreatic cyst in a further patient 13 . In another study, the resection margin was based on preoperative POP and intraductal sonography, and comparison with postoperative surgical specimens revealed only one positive resection margin (2.5 %) 14 . Miura et al. used POP with NBI to determine the extent and resection line in seven patients, with postoperative examination showing no tumor in the excision stumps 19 . In a study by Trindade et al., POP dictated the surgical plan determined prior to POP in 42 % of patients with MD-IPMN (13/31) 29 . This was more common in those with a diffusely dilated MPD > 10 mm (10/31; 77 %) compared with those with a focally dilated MPD (3/18; 17 %; *P* = 0.001).

Five studies investigated whether pre- or intraoperative pancreatoscopy findings altered the intended surgical approach, which was based on cross-sectional imaging, ERCP, and/or EUS 10 23 24 28 31 . After initial transection, IOP was performed to check the remaining PD for lesions. For POP, it was not clearly reported how the additional margin was determined. Overall, determination of the extent of the lesion or identification of skip lesions by visualization or biopsy resulted in an altered surgical approach in 13 %–62 % of patients: in 13 %–31 % of patients, it resulted in a more extensive surgical resection 10 23 24 28 31 ; in 6 %–31 % of patients, it resulted in a less extensive surgical resection 23 24 31 . Two studies reported that preoperative POP findings affected clinical decision-making or determination of the excision line, without reporting the initial surgical plan 21 22 . The specifics of these studies can be found in Table 2 and **Appendix 3 s** . Complications related to IOP were not reported.

## Discussion

The risk of malignancy in IPMN is highly variable as BD-IPMNs contain malignancy in a minority of patients, while MD-IPMNs have a higher reported incidence of malignancy 3 4 . Although the general recommendation of the International Association of Pancreatology and others is that mucin-producing neoplasms with high risk features or MD-IPMN > 10 mm should undergo surgical resection, obtaining a definite diagnosis and assessing the possible intraductal extent can often be difficult. As such, the primary utility of POP in IPMN is considered threefold: (i) to confirm the diagnosis in equivocal cases based on imaging and history, especially when there is a question of chronic pancreatitis versus IPMN; (ii) to assess the presence of malignancy or high grade dysplasia; and (iii) to map the IPMN in order to guide resection margins. In current clinical practice, the exact role of POP remains to be determined. Its use remains limited to large volume referral centers, and available data regarding its efficacy and safety are limited and heterogeneous. In this meta-analysis, we summarize the available data on the use of POP in patients with (suspected) IPMN. Following a strict predefined search strategy, we identified 25 articles eligible for inclusion.

Overall, cannulation of the MPD with the pancreatoscope was successful in the vast majority of patients in whom standard MPD access had already been was achieved (86 %–100 %), and adequate visualization of the target area could be achieved in 60 %–100 % of patients, with the vast majority of studies reporting success rates of 100 %. Predictive factors reported for failure to reach the target area were: impaired visibility due to an abundance of mucus; anatomical features such as strictures; or a nondilated MPD 11 12 22 25 .

Despite these high technical success rates, AEs occurred in 12 % of patients 11 13 14 15 20 21 22 26 27 28 29 . Because the indication for pancreatoscopy is only diagnostic, the risk of complications may more readily outweigh the benefit of the procedure in comparison with therapeutic procedures. An acknowledgement of the high risk of pancreatitis is of clinical relevance because it may lead to postponement or even deferral of surgery. However, in the majority of the patients, PEP was treated conservatively and its severity was mild to moderate. The most important risk factor for PEP in the evaluated studies was the presence of a focally or mild-to-nondilated MPD, as compared with patients with a diffusely widened MPD, in whom the incidence of PEP was lower 21 29 . All this should be interpreted in light of the observation that preoperative pancreatoscopy influenced the type/extent of surgery in the vast majority of patients with a diffusely dilated MPD, but only in less than 20 % of those with a focally dilated MPD 29 .

Several studies have investigated the different pancreatoscopic features that are consistent with benign and malignant IPMN 8 10 11 12 14 15 16 26 . Features that were more frequently identified in patients diagnosed with malignant IPMN were intraductal fish-egg lesions, prominent vascular changes, villous projections, and vegetative projections. Also, friability and a coarse mucosa were described as being related to malignancy. In addition, three studies investigated the additional value of NBI in the assessment of intraductal lesions and showed promising results, with improved visualization of the vasculature and flat lesions, along with identification of skip lesions that were otherwise not detected 17 18 19 . Subsequently, identification of these areas could improve the yield of intraductal (targeted) biopsies.

Unfortunately, there are no studies available that have investigated the interobserver agreement of the different pancreatoscopic features and therefore their exact clinical value remains unclear. Sethi et al. previously showed that interobserver agreement of visual assessment of indeterminate biliary strictures is very low, even among experienced endoscopists 34 . It is likely that this would be the case for IPMN as well.

Considering the moderate sensitivity and specificity rates reported in this review for a visual diagnosis of IPMN (64 %–100 %, and 75 %–100 %, respectively), histological confirmation remains important. The yield of POP-guided targeted biopsies and/or cytology was reported in eleven studies with widely varying results, with sensitivity rates ranging from 13 % to 100 % and specificity rates ranging from 53 % to 100 % 11 14 16 21 22 25 26 27 28 29 32 . This variation can be explained by the small biopsies obtained via POP, owing to difficult maneuvering of the biopsy forceps, which make pathological diagnosis difficult. With regard to cytology examination, different sampling methods were used and different rates were reported for irrigation fluid compared with pancreatic juice obtained via POP. Interestingly, two studies showed that samples obtained by POP showed higher diagnostic accuracy rates than samples obtained by a catheter 22 32 .

To improve the diagnostic yield of POP-guided targeted biopsies and cytology, future studies are needed to investigate the best collection method of fluid for cytology examinations and biopsy samples. To further optimize the yield of POP, there are some studies that have indicated that probe-based confocal laser endomicroscopy might be helpful in determining the nature of pancreatic lesions, such as IPMN, and its clinical management 35 36 .

The most important question in the setting of pancreatoscopy as a complementary diagnostic tool in the work-up of IPMN is its actual impact on clinical (therapeutic) management and patient outcomes. Results varied greatly between the studies, from only 13 % of patients having their surgical approach altered to almost all patients being impacted 10 13 14 19 21 22 23 24 28 29 31 . In some studies, POP detected multifocal lesions that were otherwise not detected or could have been mistaken for chronic pancreatitis 10 20 21 22 23 24 25 26 27 28 29 30 31 32 33 34 35 36 37 . The nature of the included studies makes it difficult however to determine the exact role of POP in the preoperative diagnostic work-up. Ideally, the primary utility of POP in IPMN would be to confirm the diagnosis of IPMN in persistent equivocal cases, or to map the extent of the IPMN where there is uncertainty regarding the extent of surgery. However, it should preferably only be performed after a diagnostic work-up including imaging (CT and/or MRI) and EUS with tissue acquisition, and following a multidisciplinary meeting. The Fukuoka guidelines advise performing EUS if there are worrisome features present on cross-sectional imaging 4 . However, the yield is relatively low with a pooled sensitivity of 54 % (95 %CI 49 %–59 %) and specificity of 93 % (95 %CI 90 %–95 %) for EUS-guided fine-needle aspiration 38 .

When determining the exact position of POP in the diagnostic work-up of IPMN, the risks and benefits should be weighed. As mentioned before, POP carries a considerable risk of PEP, although this is mainly mild. In addition, POP might be more costly compared with other diagnostic tools, such as CT or MRI. On the other hand, performing unnecessary or overly extensive surgery carries a risk of surgery-related AEs and is also costly. Currently, new studies on POP and IOP are underway that are also taking into account cost-effectiveness (NCT03062124 and NCT03729453), and results are eagerly awaited.

Some limitations need to be discussed. First, most studies were of a retrospective nature, did not involve a consecutive case series, and reported only descriptive data. Second, many different types of pancreatoscopes were used and, maybe more importantly, only five studies used the Spyglass DS, a digital pancreatoscope with an improved image quality compared with previous through-the-scope pancreatoscopes. In today’s clinical practice the Spyglass DS and the Spyglass DS II are the most commonly used pancreatoscopes 39 . These scopes have a wider range of view, with an enhanced image quality, which may increase the diagnostic yield of pancreatoscopy in the setting of the diagnostic work-up of IPMN. Third, as discussed in the previous paragraph, the included studies used different diagnostic work-up protocols and different outcome measurements, making it difficult to directly compare their outcomes in order to define the role of POP in the current diagnostic work-up. For all these reasons, a systematic quantitative data analysis was not possible for most outcome parameters, which prohibits the drawing of a definite conclusion regarding the role of POP in current clinical practice.

In conclusion, this is the first literature review to summarize the current knowledge on the role of POP in the diagnostic algorithm of IPMN. POP has a high technical success rate and seems to provide adequate visualization of the target area, in particular in patients with a dilated MPD. POP may be useful in the preoperative work-up for assessment of the extent and exact location of the lesion, as well as to identify the existence of skip lesions. However, despite the reasonably high diagnostic accuracy rates that have been reported, the exact role of POP in the diagnostic work-up still remains unclear, mostly because of methodological shortcomings and heterogeneity between studies. Large multicenter consecutive prospective studies performed according to a predefined protocol, including well-described procedural aspects, imaging documentation (preferably by video), and the application of intraductal pancreatoscopy-guided biopsies, are needed to better define the role of POP in the diagnostic algorithm of IPMN.

## Supplementary material

Supplementary material Zusatzmaterial
