# Supplementary material for: The role of pancreatoscopy in the diagnostic work-up of intraductal papillary mucinous neoplasms: a systematic review and meta-analysis
Source: Endoscopy. 2022 Jul 20;55(1):25–35. doi: 10.1055/a-1869-0180 (PMC9767751; doi:10.1055/a-1869-0180)
Supplement: Supplementary file 3 — Supplementary material [file 21565supmat_10-1055-a-1869-0180.pdf]

Supplementary material

The role of pancreatoscopy in the diagnostic work-up of intraductal papillary mucinous neoplasms: a systematic review and meta-analysis

David M. de Jong, Pauline M. C. Stassen, Bas Groot Koerkamp, Mark Ellrichmann, Petko I. Karagyozov, Andrea Anderloni, Lenna Kylänpää, George J. M. Webster, Lydi M. J. W. van Driel, Marco J. Bruno, Pieter J. F. de Jonge

Table 1s PRISMA checklist

Section and Topic	Item #	Checklist item	Page number where item is reported
**TITLE**			
Title	1	Identify the report as a systematic review.	1
**ABSTRACT**			
Abstract	2	See the PRISMA 2020 for Abstracts checklist.	2
**INTRODUCTION**			
Rationale	3	Describe the rationale for the review in the context of existing knowledge.	3
Objectives	4	Provide an explicit statement of the objective(s) or question(s) the review addresses.	3
**METHODS**			
Eligibility criteria	5	Specify the inclusion and exclusion criteria for the review and how studies were grouped for the syntheses.	3-4
Information sources	6	Specify all databases, registers, websites, organisations, reference lists and other sources searched or consulted to identify studies. Specify the date when each source was last searched or consulted.	3-4
Search strategy	7	Present the full search strategies for all databases, registers and websites, including any filters and limits used.	3-4, Suppl
Selection process	8	Specify the methods used to decide whether a study met the inclusion criteria of the review, including how many reviewers screened each record and each report retrieved, whether they worked independently, and if applicable, details of automation tools used in the process.	4
Data collection process	9	Specify the methods used to collect data from reports, including how many reviewers collected data from each report, whether they worked independently, any processes for obtaining or confirming data from study investigators, and if applicable, details of automation tools used in the process.	4
Data items	10a	List and define all outcomes for which data were sought. Specify whether all results that were compatible with each outcome domain in each study were sought (e.g. for all measures, time points, analyses), and if not, the methods used to decide which results to collect.	4
	10b	List and define all other variables for which data were sought (e.g. participant and intervention characteristics, funding sources). Describe any assumptions made about any missing or unclear information.	4

Supplementary material

Section and Topic	Item #	Checklist item	Page number where item is reported
Study risk of bias assessment	11	Specify the methods used to assess risk of bias in the included studies, including details of the tool(s) used, how many reviewers assessed each study and whether they worked independently, and if applicable, details of automation tools used in the process.	n.a.
Effect measures	12	Specify for each outcome the effect measure(s) (e.g. risk ratio, mean difference) used in the synthesis or presentation of results.	4
Synthesis methods	13a	Describe the processes used to decide which studies were eligible for each synthesis (e.g. tabulating the study intervention characteristics and comparing against the planned groups for each synthesis (item #5)).	4
	13b	Describe any methods required to prepare the data for presentation or synthesis, such as handling of missing summary statistics, or data conversions.	4
	13c	Describe any methods used to tabulate or visually display results of individual studies and syntheses.	4
	13d	Describe any methods used to synthesize results and provide a rationale for the choice(s). If meta-analysis was performed, describe the model(s), method(s) to identify the presence and extent of statistical heterogeneity, and software package(s) used.	4
	13e	Describe any methods used to explore possible causes of heterogeneity among study results (e.g. subgroup analysis, meta-regression).	n.a.
	13f	Describe any sensitivity analyses conducted to assess robustness of the synthesized results.	4
Reporting bias assessment	14	Describe any methods used to assess risk of bias due to missing results in a synthesis (arising from reporting biases).	n.a.
Certainty assessment	15	Describe any methods used to assess certainty (or confidence) in the body of evidence for an outcome.	n.a.
**RESULTS**			
Study selection	16a	Describe the results of the search and selection process, from the number of records identified in the search to the number of studies included in the review, ideally using a flow diagram.	5, Fig 1
	16b	Cite studies that might appear to meet the inclusion criteria, but which were excluded, and explain why they were excluded.	n.a.
Study characteristics	17	Cite each included study and present its characteristics.	5, Fig 2
Risk of bias in studies	18	Present assessments of risk of bias for each included study.	n.a.
Results of individual studies	19	For all outcomes, present, for each study: (a) summary statistics for each group (where appropriate) and (b) an effect estimate and its precision (e.g. confidence/credible interval), ideally using structured tables or plots.	5-8, Fig 2
Results of syntheses	20a	For each synthesis, briefly summarise the characteristics and risk of bias among contributing studies.	n.a.
	20b	Present results of all statistical syntheses conducted. If meta-analysis was done, present for each the summary estimate and its precision (e.g. confidence/credible interval) and measures of statistical heterogeneity. If comparing groups, describe the direction of the effect.	5, Fig 2 & 4
	20c	Present results of all investigations of possible causes of heterogeneity among study results.	n.a.
	20d	Present results of all sensitivity analyses conducted to assess the robustness of the synthesized results.	n.a.
Reporting biases	21	Present assessments of risk of bias due to missing results (arising from reporting biases) for each synthesis assessed.	n.a.

Supplementary material

Section and Topic	Item #	Checklist item	Page number where item is reported
Certainty of evidence	22	Present assessments of certainty (or confidence) in the body of evidence for each outcome assessed.	n.a.
**DISCUSSION**			
Discussion	23a	Provide a general interpretation of the results in the context of other evidence.	9-11
	23b	Discuss any limitations of the evidence included in the review.	9-11
	23c	Discuss any limitations of the review processes used.	9-11
	23d	Discuss implications of the results for practice, policy, and future research.	9-11
**OTHER INFORMATION**			
Registration and protocol	24a	Provide registration information for the review, including register name and registration number, or state that the review was not registered.	n.a.
	24b	Indicate where the review protocol can be accessed, or state that a protocol was not prepared.	n.a.
	24c	Describe and explain any amendments to information provided at registration or in the protocol.	n.a.
Support	25	Describe sources of financial or non-financial support for the review, and the role of the funders or sponsors in the review.	12
Competing interests	26	Declare any competing interests of review authors.	12
Availability of data, code and other materials	27	Report which of the following are publicly available and where they can be found: template data collection forms; data extracted from included studies; data used for all analyses; analytic code; any other materials used in the review.	n.a.

From: Page MJ, McKenzie JE, Bossuyt PM, Boutron I, Hoffmann TC, Mulrow CD, et al. The PRISMA 2020 statement: an updated guideline for reporting systematic reviews. BMJ 2021;372:n71. doi: 10.1136/bmj.n71

For more information, visit: http://www.prisma-statement.org/

Supplementary material

Table 2s. Full search strategy and results on 11th of February 2022

Database	Search strategy	N
EMBASE (via Pubmed)	('intraductal papillary mucinous tumor'/de OR 'pancreas intraductal papillary mucinous tumor'/de OR (((intraductal* OR intra-ductal*) NEAR/10 (papill* OR pancrea*) NEAR/10 (tumo* OR neopla* OR carcinoma* OR adenocarcinoma* OR adenoma* OR cystadeno* OR lesion*)) OR ((pancrea*) NEAR/10 (intrapapill* OR intra-papill*) NEAR/10 (tumo* OR neopla* OR carcinoma* OR adenocarcinoma* OR adenoma* OR cystadeno* OR lesion*)) OR ipmn OR ipmt OR ipmc OR ipma OR ipmns OR ipmts OR ipmcs OR ipmas):ab,ti) AND ('cholangiopancreatoscopy'/de OR 'pancreatoscopy'/de OR 'pancreatoscope'/de OR 'peroral pancreatoscopy'/de OR pancreatography/de OR 'single operator peroral pancreatoscope'/de OR 'single operator peroral pancreatoscopy'/de OR 'intraoperative pancreatoscopy'/de OR (cholangiopancreatoscop* OR pancreatoscop* OR spyglass* OR spy OR Pancreaticocholangioscop* OR pancreatograph*):ab,ti) NOT ([Conference Abstract]/lim)	322
Medline Ovid	(Pancreatic Intraductal Neoplasms/ OR (((intraductal* OR intra-ductal*) ADJ10 (papill* OR pancrea*) ADJ10 (tumo* OR neopla* OR carcinoma* OR adenocarcinoma* OR adenoma* OR cystadeno* OR lesion*)) OR ((pancrea*) ADJ10 (intrapapill* OR intra-papill*) ADJ10 (tumo* OR neopla* OR carcinoma* OR adenocarcinoma* OR adenoma* OR cystadeno* OR lesion*)) OR ipmn OR ipmt OR ipmc OR ipma OR ipmns OR ipmts OR ipmcs OR ipmas).ab,ti.) AND ((cholangiopancreatoscop* OR pancreatoscop* OR spyglass* OR spy OR Pancreaticocholangioscop* OR pancreatograph*).ab,ti.) NOT (news OR congres* OR abstract* OR book* OR chapter* OR dissertation abstract*).pt.	185
Web of science	TS=(((((intraductal* OR intra-ductal*) NEAR/10 (papill* OR pancrea*) NEAR/10 (tumo* OR neopla* OR carcinoma* OR adenocarcinoma* OR adenoma* OR cystadeno* OR lesion*)) OR ((pancrea*) NEAR/10 (intrapapill* OR intra-papill*) NEAR/10 (tumo* OR neopla* OR carcinoma* OR adenocarcinoma* OR adenoma* OR cystadeno* OR lesion*)) OR ipmn OR ipmt OR ipmc OR ipma OR ipmns OR ipmts OR ipmcs OR ipmas)) AND ((cholangiopancreatoscop* OR pancreatoscop* OR spyglass* OR spy OR Pancreaticocholangioscop* OR pancreatograph*))) AND DT=(article)	158
Cochrane CENTRAL	((((intraductal* OR intra next ductal*) NEAR/10 (papill* OR pancrea*) NEAR/10 (tumo* OR neopla* OR carcinoma* OR adenocarcinoma* OR adenoma* OR cystadeno* OR lesion*)) OR ((pancrea*) NEAR/10 (intrapapill* OR intra next papill*) NEAR/10 (tumo* OR neopla* OR carcinoma* OR adenocarcinoma* OR adenoma* OR cystadeno* OR lesion*)) OR ipmn OR ipmt OR ipmc OR ipma OR ipmns OR ipmts OR ipmcs OR ipmas):ab,ti) AND ((cholangiopancreatoscop* OR pancreatoscop* OR spyglass* OR spy OR Pancreaticocholangioscop* OR pancreatograph*):ab,ti)	3
Google scholar	"intraductal|ductal papillary|pancreas|pancreatic tumor|neoplasm|tumors|neoplasms" cholangiopancreatoscopy|pancreatoscopy|spyglass|Pancreaticocholangioscopy	100

Supplementary material

Fig. 1s Pooled post-ERCP pancreatitis rate: all studies that reported post-ERCP pancreatitis

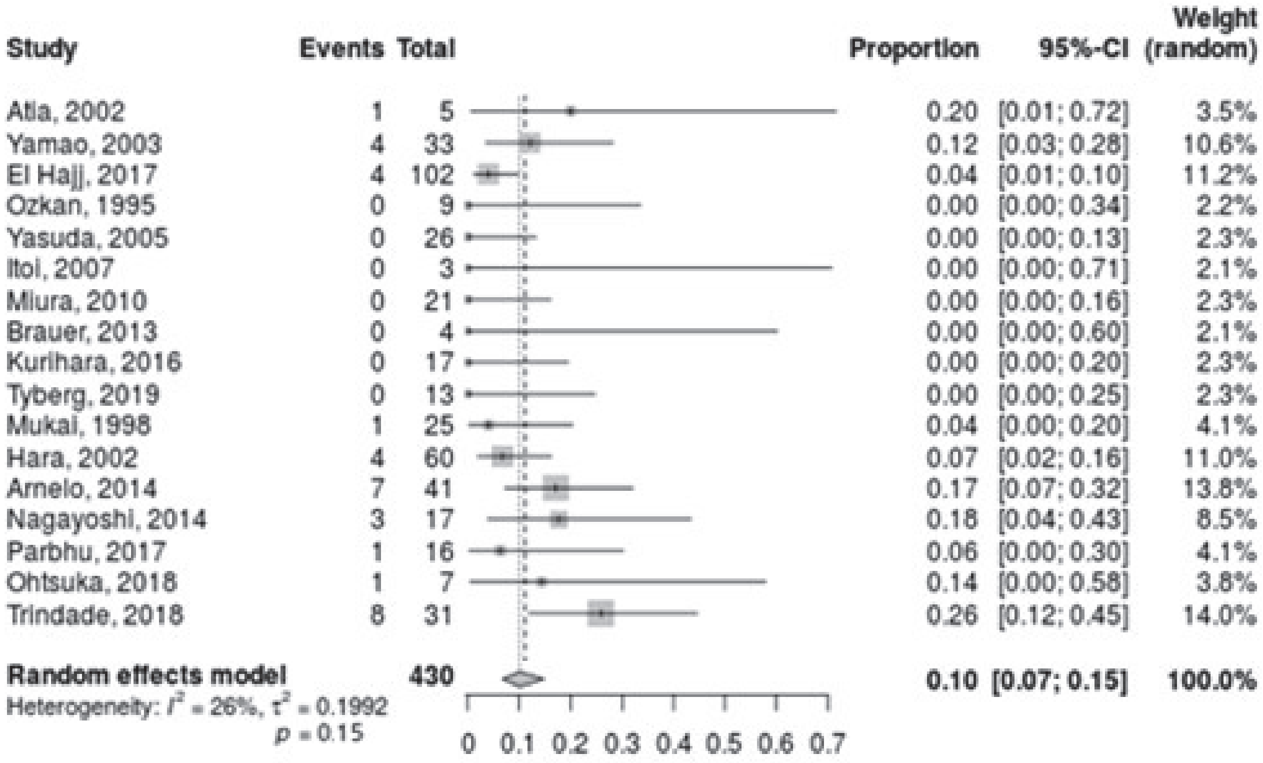

Supplementary material

Appendix 1s Narrow-band imaging

Miura et al. assessed POP-guided NBI in 21 patients [1]. Vascular patterns and protrusions were detected more clearly as compared to white light. Similar to this, Itoh et al. also described that in all five patients (100%) NBI was able to visualize small vessels and the superficial architecture, providing good delineation of IPMN [2]. Finally, in a study by Itoi et al., including three patients, NBI showed fine capillary vessels in all three patients, that were otherwise not detected by using white light only, resulting in detection of skip tumor lesions in the tail of the pancreas in one patient and detection of a tumor at the transition site of head to body in another [3].

Appendix 2s Diagnostic value

A Targeted biopsies and cytology obtained using POP

Six studies investigated the diagnostic accuracy of targeted biopsies taken under direct pancreatoscopic visualization, for differentiation between malignant and non-invasive disease in patients with IPMN [4-9]. Results are shown in **Table 1**. Kurihara *et al.* reported that adequate tissue samples could be obtained in 10/11 patients (91%) with suspicion of MD-IPMN [10]. Ohtsuka *et al*. found a sensitivity of 0% for detecting HGD [8]. During the preoperative work-up targeted biopsies were taken from the lesion of interest, but showed no HGD contrary to the surgical specimens of three patients. El Hajj *et al*. found that POP-guided biopsies in addition to visualization alone improved the sensitivity (87% to 91%), specificity (86% to 95%), positive predictive value (83% to 94%), negative predictive value (91% to 93%), and accuracy (87% to 94%) [11], in detecting pancreatic duct neoplasia, both in patients with and without IPMN. Parbhu *et al*. reported a 64% sensitivity and 100% specificity of POP-guided biopsies in correctly diagnosing IPMN [12]. Results regarding pancreatic juice collection by POP are presented in **Table 1**.

B Pancreatic juice collection by POP

Seven studies investigated the diagnostic characteristics of pancreatic fluid cytology directly obtained via pancreatoscopy. Presence of malignancy or high suspicion of malignancy were indicative of malignant IPMN. Sensitivity rates ranged between 13-100% and specificity rates were 100% [4-8, 13, 14]. Two studies reported on molecular markers. No studies reported on the use of secretin. Of note, different rates are reported for cytology on pancreatic juice and on irrigation fluid analysis. Four studies reported on the use of cytological analysis on irrigation fluid, with sensitivity rates between 33-100% [4, 7, 8], specificity of 100% [4, 7], and accuracy of 89% [4]. In addition, Arnelo *et al*. reported that cytology results showed malignancy in one of the 22 (out of 41) patients in whom irrigation fluid cytology was performed [6]. Important to note is that in situ hybridization (FISH) was used in 3/41 patients and DNA flow cytometry in 27/41. Finally, three studies reported on diagnostic characteristics of pancreatic juice cytology [5, 13, 14]. According to these studies sensitivity ranged from 15-67% [5, 13, 14] and specificity was 100% [5, 13, 14], with an overall diagnostic accuracy of 44% [13]. Hara et al. determined the presence of the K-ras point mutation in the pancreatic juice. Yamaguchi *et al*. compared the diagnostic accuracy of pancreatic juice cytology collected by POP or by catheter and

Supplementary material

found a higher sensitivity, but not statistically significant, for pancreatic juice cytology collected by POP as compared to catheter-assisted aspiration (68% versus 38%, respectively, p=0.055) [14].

A study by Uehara *et al*. who investigated the diagnostic value of pancreatic cytology in diagnosing carcinoma in situ, showed that in all eleven patients cancerous cells were found on examination of pancreatic fluid aspirated through the pancreatoscope [15]. These findings suggest that pancreatic juice sampling via POP should be considered for cytopathological examination, particularly in cases in which the EUS-fine needle aspiration was inconclusive or not possible. Because pancreatic juice cytology collection is time consuming, preselection of patients with high suspicion of malignancy can be considered.

Appendix 3s Determination of the extent of IPMN by pancreatoscopy

In a retrospective study from Ohtsuka *et al.* pancreatoscopy changed the surgical management only in 1/7 patients (14%) and failed to detect 1 concomitant ductal adenocarcinoma [8]. Kaneko *et al.* found that IOP detected 10 cases of IPMN that were not detected by EUS or ERCP. Half of them (5/10) were intraductal multicentric lesions. Finally, in 3/5 patients (60%) an additional pancreatic resection was performed, resulting in an altered surgical plan in 13% of patients overall [16]. Pucci *et al*. reported that in 8/23 patients (35%) final resection margins were influenced by pancreatoscopic findings. With regards to patients with suspected IPMN (n=18), pancreatoscopy identified six additional lesions in 6 patients, after which in five patients (29%) the resection margin was extended and in one patient (6%) margin was spared [17]. In two patients adenocarcinoma was removed with the extended margin, in one patient with HGD, in one patient with moderate dysplasia and in one patient with a BD-IPMN with LGD. In the patient with less extensive margin LGD was found in the neck. Navez et al. reported that IOP and intraductal biopsies modified initial planned surgical resection in 23.8% of the 21 patients [18]. POP-guided biopsies may also be helpful in determining the resection margins intraoperatively. Tyberg *et al*. reported that in 7 of the 8 patients in whom the surgical plan was changed, this was based on visual findings in combination with results from targeted biopsies [19]. In 2/8 patients in whom the surgical plan was altered on POP findings, from total pancreatectomy to pancreatoduodenectomy, post-operative resection margins were positive.

Regarding the two studies not reporting prior surgical plan, Arnelo *et al*. reported that additional information was provided by POP in 39 out of 41 patients (95%) with suspected IPMN while affecting clinical decision making in 76% [6]. According to Nagayoshi *et al*., in 3/17 patients (18%) POP was useful in determining the excision line. In the three patients in whom POP contributed to determination of the excision line, a transition of fish-egg like lesions to normal mucosa could be clearly identified [7].

Supplementary material

References

1. Miura T, Igarashi Y, Okano N, et al. Endoscopic diagnosis of intraductal papillary-mucinous neoplasm of the pancreas by means f peroral pancreatoscopy using a small-diameter videoscope and narrow-band imaging. Dig Endosc. 2010;22(2):119-23.
2. Itoh A, Hirooka Y, Kawashima H, et al. Endoscopic approach to the pancreatobiliary tract using narrow band imaging. Digestive Endoscopy. 2007;19:S115-S20.
3. Itoi T, Sofuni A, Itokawa F, et al. Initial experience of peroral pancreatoscopy combined with narrow-band imaging in the diagnosis of intraductal papillary mucinous neoplasms of the pancreas (with videos){A figure is presented}. Gastrointest Endosc. 2007;66(4):793-7.
4. Mukai H, Yasuda K, Nakajima M. Differential diagnosis of mucin-producing tumors of the pancreas by intraductal ultrasonography and peroral pancreatoscopy. Endoscopy. 1998;30 Suppl 1:A99-102.
5. Yasuda K, Sakata M, Ueda M, et al. The use of pancreatoscopy in the diagnosis of intraductal papillary mucinous tumor lesions of the pancreas. Clin Gastroenterol Hepatol. 2005;3(7 SUPPL.):S53-S7.
6. Arnelo U, Siiki A, Swahn F, et al. Single-operator pancreatoscopy is helpful in the evaluation of suspected intraductal papillary mucinous neoplasms (IPMN). Pancreatology. 2014;14(6):510-4.
7. Nagayoshi Y, Aso T, Ohtsuka T, et al. Peroral pancreatoscopy using the SpyGlass system for the assessment of intraductal papillary mucinous neoplasm of the pancreas. J Hepato-Biliary-Pancreatic Sci. 2014;21(6):410-7.
8. Ohtsuka T, Gotoh Y, Nakashima Y, et al. Role of SpyGlass-DStm in the preoperative assessment of pancreatic intraductal papillary mucinous neoplasm involving the main pancreatic duct. Pancreatology. 2018;18(5):566-71.
9. Trindade AJ, Benias PC, Kurupathi P, et al. Digital pancreatoscopy in the evaluation of main duct intraductal papillary mucinous neoplasm: A multicenter study. Endoscopy. 2018;50(11):1095-8.
10. Kurihara T, Yasuda I, Isayama H, et al. Diagnostic and therapeutic single-operator cholangiopancreatoscopy in biliopancreatic diseases: Prospective multicenter study in Japan. World J Gastroenterol. 2016;22(5):1891-901.
11. El Hajj II, Brauer BC, Wani S, et al. Role of per-oral pancreatoscopy in the evaluation of suspected pancreatic duct neoplasia: a 13-year U.S. single-center experience. Gastrointest Endosc. 2017;85(4):737-45.
12. Parbhu SK, Siddiqui AA, Murphy M, et al. Efficacy, Safety, and Outcomes of Endoscopic Retrograde Cholangiopancreatography with Per-Oral Pancreatoscopy: A Multicenter Experience. J Clin Gastroenterol. 2017;51(10):e101-e5.
13. Hara T, Yamaguchi T, Ishihara T, et al. Diagnosis and patient management of intraductal papillary-mucinous tumor of the pancreas by using peroral pancreatoscopy and intraductal ultrasonography. Gastroenterology. 2002;122(1):34-43.
14. Yamaguchi T, Shirai Y, Ishihara T, et al. Pancreatic juice cytology in the diagnosis of intraductal papillary mucinous neoplasm of the pancreas: Significance of sampling by peroral pancreatoscopy. Cancer. 2005;104(12):2830-6.
15. Uehara H, Nakaizumi A, Tatsuta M, et al. Diagnosis of carcinoma in situ of the pancreas by peroral pancreatoscopy and pancreatoscopic cytology. Cancer. 1997;79(3):454-61.
16. Kaneko T, Nakao A, Nomoto S, et al. Intraoperative pancreatoscopy with the ultrathin pancreatoscope for mucin-producing tumors of the pancreas. Archives of …. 1998.
17. Pucci MJ, Johnson CM, Punja VP, et al. Intraoperative pancreatoscopy: a valuable tool for pancreatic surgeons? J Gastrointest Surg. 2014;18(6):1100-7.
18. Navez J, Hubert C, Gigot JF, et al. Impact of Intraoperative Pancreatoscopy with Intraductal Biopsies on Surgical Management of Intraductal Papillary Mucinous Neoplasm of the Pancreas. J Am Coll Surg. 2015;221(5):982-7.
19. Tyberg A, Raijman I, Siddiqui A, et al. Digital Pancreaticocholangioscopy for Mapping of Pancreaticobiliary Neoplasia: Can We Alter the Surgical Resection Margin? J Clin Gastroenterol. 2019;53(1):71-5.
